# Endovascular stenting using a sagittal sinus approach for sigmoid sinus wall dehiscence related to intractable pulsatile tinnitus: a case series

**DOI:** 10.1186/s13256-024-04591-3

**Published:** 2024-06-01

**Authors:** Luis Alberto Ortega-Porcayo, Guillermo Gonzalez-Garibay, Ángel Lee, Juan A. Ponce-Gómez, Victor Alcocer-Barradas, Samuel Romano-Feinholz, Marco Antonio Zenteno Castellanos

**Affiliations:** 1 Medical School, Universidad Anáhuac, Mexico City, Mexico; 2 Neurosurgery Department, Hospital Ángeles del Pedregal, Mexico City, Mexico; 3 Neurosurgery Department, Centro Médico ABC, Mexico City, Mexico; 4 Research Department, Hospital General Manuel Gea González, Mexico City, Mexico; 5 Neurosurgery Department, Instituto Nacional de Neurología y Neurocirugía, Mexico City, Mexico; 6 Neuroendovascular Therapy Department, Instituto Nacional de Neurología y Neurocirugía, Mexico City, Mexico; 7 Neuroendovascular Therapy, Hospital Ángeles del Pedregal, Mexico City, Mexico

**Keywords:** Pulsatile tinnitus, Resurfacing endovascular technique, Sagittal sinus, Sigmoid sinus dehiscence, Venous stenting

## Abstract

**Background:**

Sigmoid sinus wall dehiscence can lead to pulsatile tinnitus with a significant decrease in quality of life, occasionally leading to psychiatric disorders. Several surgical and endovascular procedures have been described for resolving dehiscence. Within endovascular procedures, the sagittal sinus approach could be a technical alternative for tracking and accurate stent positioning within the sigmoid sinus when the jugular bulb anatomy is unfavorable.

**Case presentation:**

A retrospective case series of three patients with pulsatile tinnitus due to sigmoid sinus wall dehiscence without intracranial hypertension was reviewed from January 2018 to January 2022. From the participants enrolled, the median age was 50.3 years (range 43–63), with 67% self-identifying as female and 33% as male. They self-identified as Hispanic. Sigmoid sinus dehiscence was diagnosed using angiotomography, and contralateral transverse sinus stenosis was observed in all patients. Patients underwent surgery via a navigated endovascular sagittal sinus approach for sigmoid sinus stenting. No neurological complications were associated with the procedure. Pulsatile tinnitus improved after the procedure in all patients.

**Conclusions:**

Superior sagittal sinus resection for sigmoid sinus wall stenting is a safe and effective technique. Pulsatile tinnitus due to sigmoid sinus wall dehiscence could be treated using the endovascular resurfacing stenting technique. However, further research is needed to evaluate the potential benefit of contralateral stenting for removing sinus dehiscence when venous stenosis is detected. However, resurfacing sigmoid sinus wall dehiscence results in symptomatic improvement.

## Background

Pulsatile tinnitus (PT) is a distressing symptom characterized by the perception of a rhythmic noise that resembles a heartbeat, despite the absence of any external acoustic stimulus. It can potentially lead to psychiatric disorders and affects approximately 4–10% of the general population [1–3]. Studies have shown that 4–20% of PT cases involve sigmoid sinus (SS) wall anomalies and venous system ipsilateral dominance [4–6]. These anomalies can range from a very thin and intact SS bone wall to a diverticulum with a focal protrusion into the mastoid air cells, ectasia with smooth bulging of the sinus into the mastoid air cells, or even dehiscence of the SS wall [7].

Sigmoid sinus wall dehiscence (SSWD) is an anomaly that occurs when the bone surrounding the sinus is incomplete, leading to contact between the mastoid cells and the SS. This can cause disruption of laminar flow, creating audible turbulence, increasing conduction of normal blood flow, and leading to PT [4, 8].

Treatment for PT associated with venous etiologies not associated with idiopathic intracranial hypertension may be based on resurfacing and reconstruction of SS wall anomalies. The goal of this treatment is to eliminate the sound caused by vascular turbulence and decrease the transmission of vibrations through mastoid air cells. Endovascular management of PT was first described in 2000 by Houdart with coiling and in 2004 by Zenteno with stenting [9, 10]. In the present study, we report a series of patients with PT related to ipsilateral SSWD treated using stent resurfacing endovascular technique through a neuro-navigated superior sagittal sinus (SSS) approach.

## Methodology

We conducted a retrospective case series review from January 2018 to January 2022. All patients provided informed consent according to ethical standards. The present study evaluated patient characteristics, computed tomography angiography (CTA) and digital subtraction angiography (DSA), and clinical outcomes as evaluated by a neurosurgeon and a neuroendovascular therapist. The study included three patients who had symptomatic incoercible PT related to SSWD not associated with idiopathic intracranial hypertension and were referred by otolaryngologists.

### Venous sinus stenting through a neuronavigated sagittal sinus approach

**Fig. 1:**
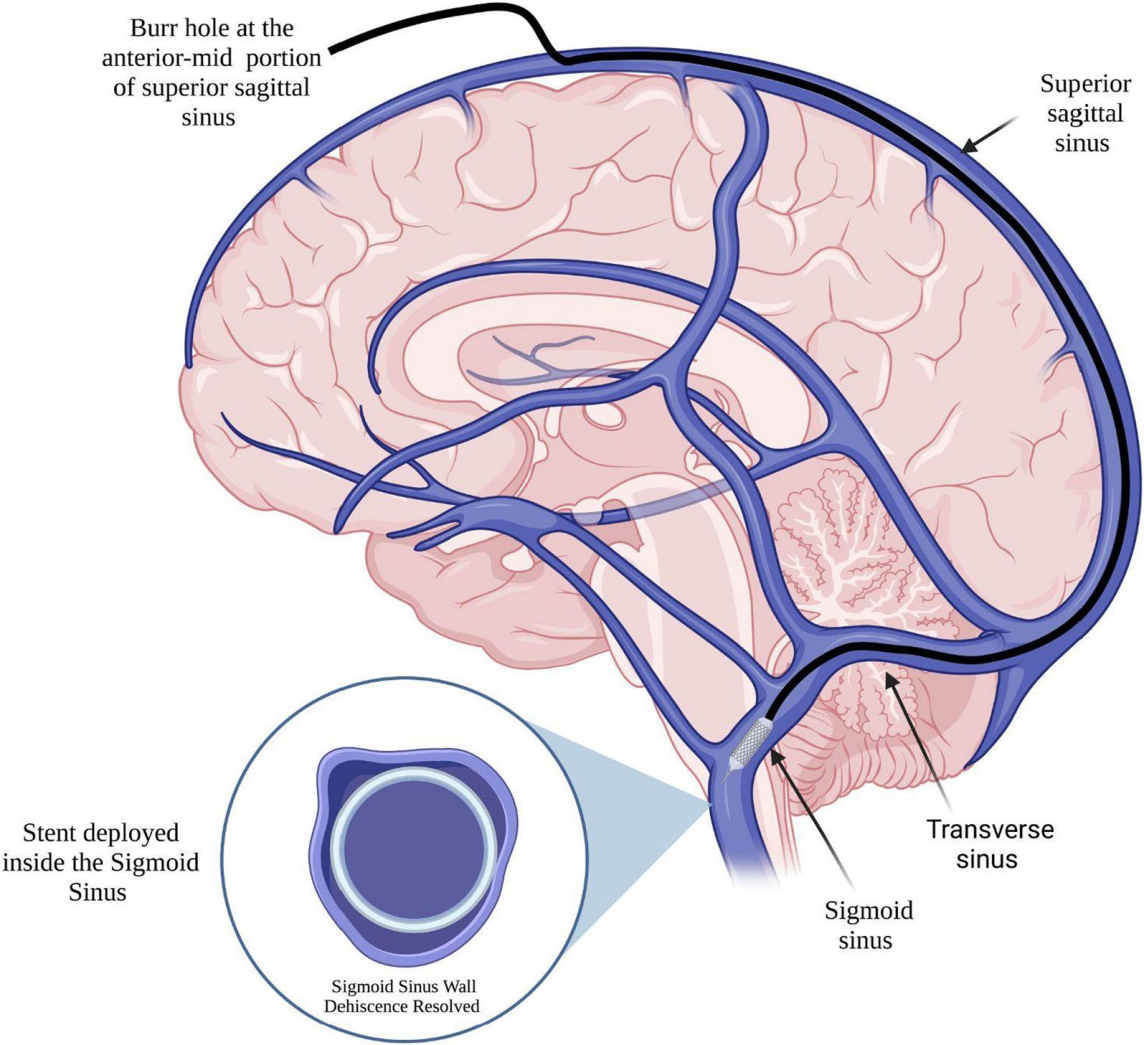
Endovascular neurosurgical approach to the sigmoid sinus involving the use of a burr hole in the anterior skull to access the superior sagittal sinus. Wall stents were advanced over the wire and deployed in the sigmoid sinus to restore wall integrity

First, a neuronavigated (Fiagon®, Hennigsdorf, Germany) burr hole was made at the anterior-mid portion of the SSS behind the hairline (Fig. 1). Access was obtained via the SSS, and a 6 F Chaperon (MicroVention, Aliso Viejo, CA) guiding catheter was positioned in the distal sigmoid sinus at the jugular bulb through a Terumo 6 F (Terumo Interventional®, Tolyo, Japan) radial introducer sheath. Then, a 7 × 50 self-expandable carotid wallstent (Boston Scientific, Marlborough, MA) was advanced over the wire and deployed in the SS, resurfacing the wall dehiscence areas. Tirofiban was administered immediately before stent deployment without previous antiplatelet medication. The entire system was removed, and SSS bleeding was controlled using absorbable hemostatic gelatin (Gelfoam®, Pfizer, NY, USA) and gentle pressure for 5 minutes. Finally, the burr hole was closed using a preshaped round plate (KLS Martin, Tuttlingen, Germany), and the fascia and skin were closed using Vycril-0 and staples, respectively (Fig. 1).

## Case presentation

### Patient 1

**Fig. 2:**
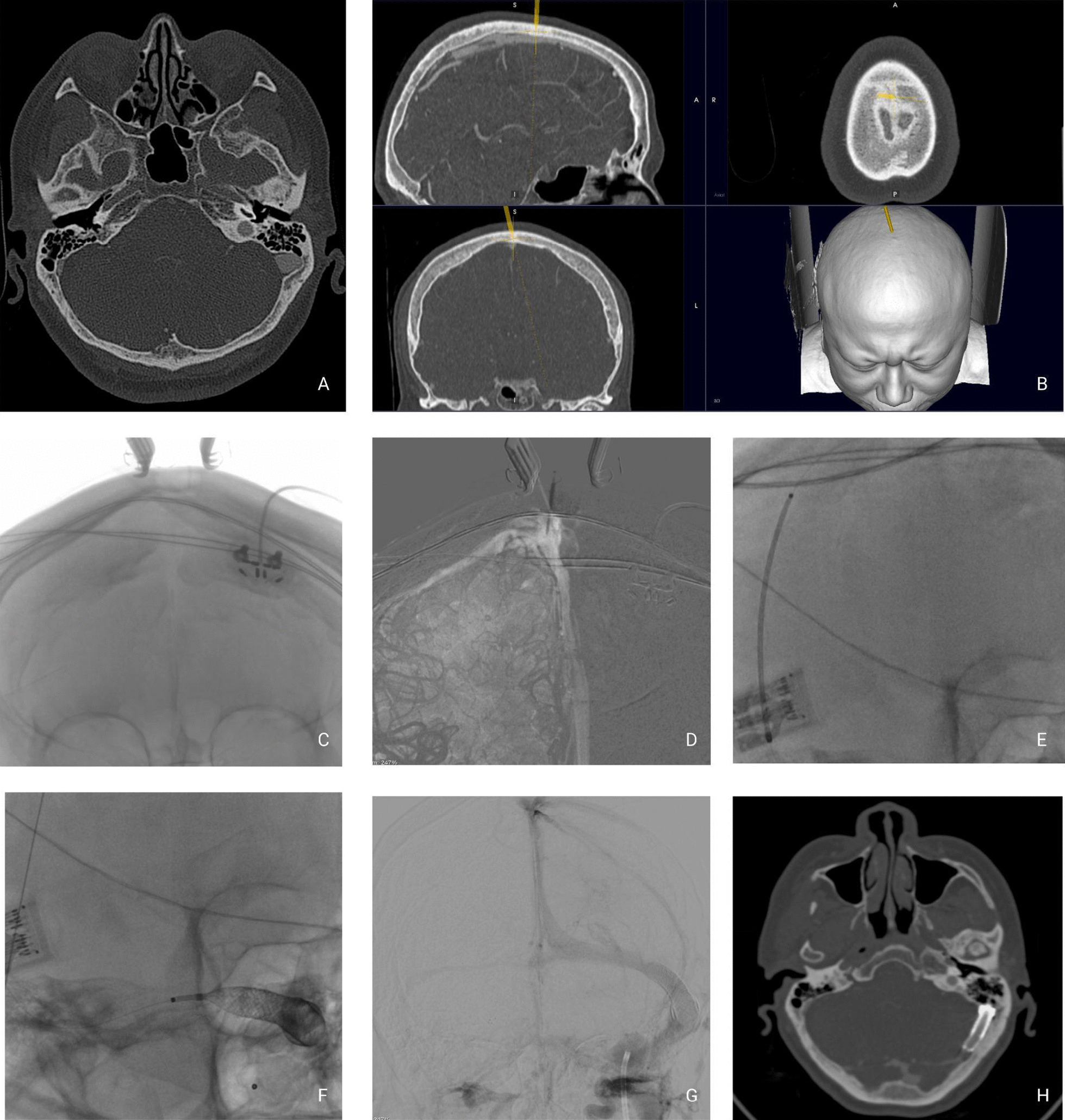
Case 1 imaging studies. **A** Preoperative CTA. **B** Neuronavigation of the SS. **C**–**E** Transoperative imaging studies of the wire advancing through the cerebral venous system to the left SS. **F**, **G** Stent deployment in the left SS lumen. **H** Postoperative CTA image showing the stent deployed in the left SS lumen

A 63-year-old Hispanic woman complained of persistent, incoercible, left-sided, pulsatile, loud, low-pitch tinnitus in her left ear for 3 months, which was exacerbated at night and disrupted her normal life. She described difficulty concentrating on certain tasks in quiet environments. She had a clinical history of major depression and chronic lumbar pain. A neurological examination, including fundoscopy, was unremarkable. The PT diminished under ipsilateral neck internal jugular vein (IJV) compression, and her audiogram was normal bilaterally. CTA showed left SSWD (Fig. 2) and contralateral transverse sinus stenosis. An incidental posterior communicating aneurysm was observed. We performed SS resurfacing venous stenting and treated the small aneurysm using the sole stenting technique [11]. The patient’s tinnitus diminished significantly after the surgery and was only noticed at night. One year after surgery, the patient reported that her tinnitus was seldomly perceived at night, and she described a significant improvement in her quality of life. Four years after surgery, the patient was asymptomatic without PT.

### Patient 2

**Fig. 3:**
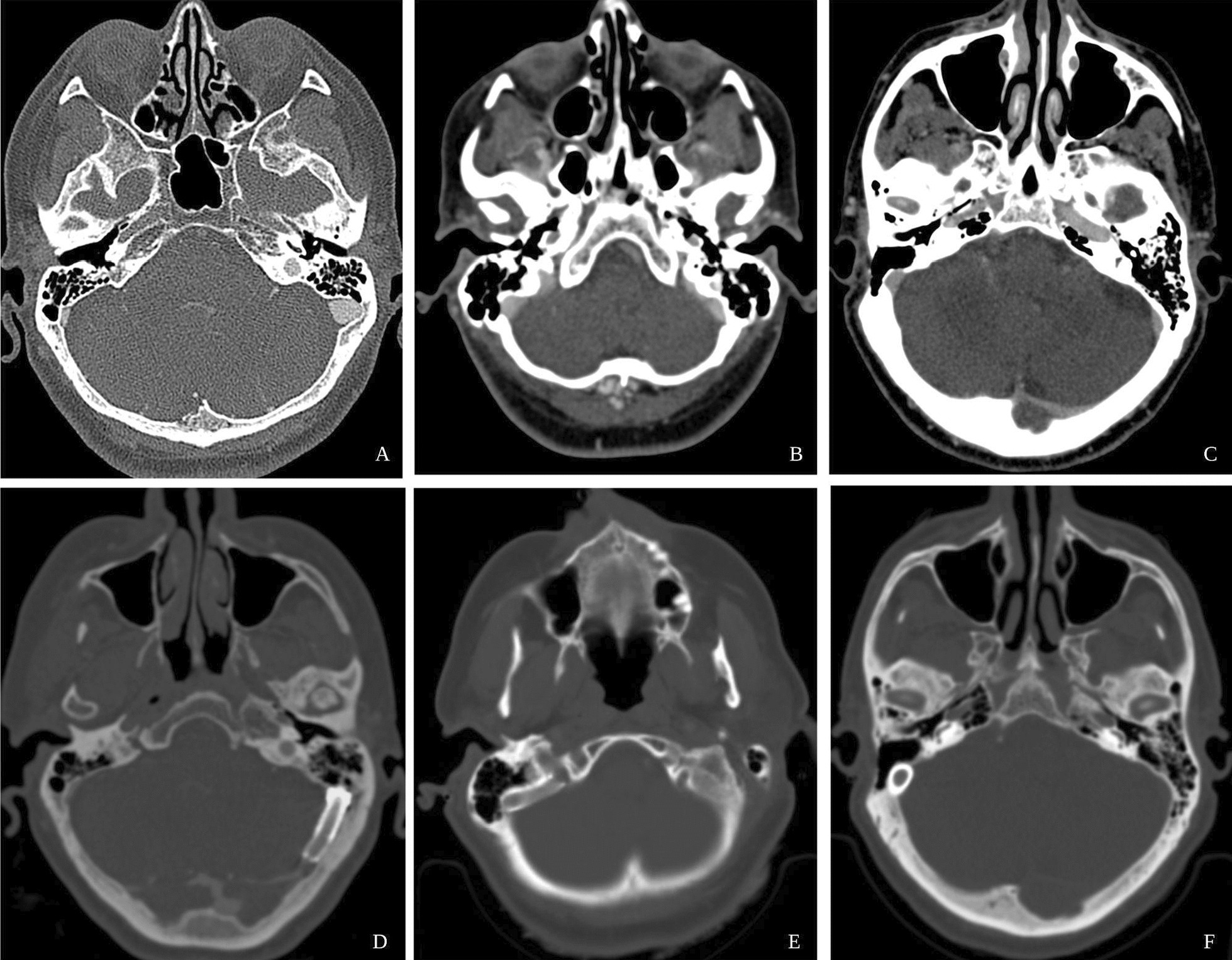
Preoperative and postoperative CTA images of patients 1, 2, and 3. **A** Patient 1 preoperative left SS dehiscence. **B** Patient 2 preoperative right SS dehiscence. **C** Patient 3: preoperative right SS dehiscence. **D** Patient 1 postoperative stent was deployed in the left SS. **E** Patient 2 postoperative stent was deployed in the right SS. **F** Patient 3 with a postoperative stent deployed in the right SS

A 43-year-old Hispanic woman presented with a 2-year history of intermittent right-sided pulsatile, loud, low-pitched tinnitus. The tinnitus coincided with her heartbeat, worsened in quiet environments, and increased during the night, disrupting her normal sleep. Neurological examination revealed mild instability and dizziness. Fundoscopy was normal, there were no focal deficits, and ipsilateral IJV compression decreased the intensity of the PT. Her bilateral audiogram was normal, and her CTA showed right SSWD (Fig. 3) and contralateral transverse sinus stenosis. SS resurfacing and venous stenting were performed, and the patient experienced immediate improvement after surgery. A total of 1-year post-surgery, the patient occasionally reported perceiving pulsatile, soft, low-pitched tinnitus at night but reported a significant improvement in her quality of life and sleep.

### Patient 3

A 45-year-old Hispanic man presented with a history of right hypoacusis, dizziness, clogged ears, and continuous intractable right pulsatile, loud, high-pitched tinnitus. The patient had previously undergone surgical reconstruction of the transmastoid SS wall. Neurological examination and fundoscopy were normal. Cranial eighth nerve examination revealed right sensorineural hypoacusis, with left Weber and bilateral positive Rinne tests. Changes in PT density were observed under ipsilateral IJV compression. An audiogram demonstrated severe right sensorineural hypoacusis and left sensorineural hypoacusis limited to high frequencies. CTA showed right SSWD (Fig. 3) and contralateral transverse sinus stenosis. SS resurfacing and venous stenting were performed, and the patient experienced mild improvement after surgery. However, he had a skin surgical site infection eight days post-surgery, which was resolved by a 1-week treatment with local neomycin ointment and amoxicillin/clavulanic acid, with no further complications. A total of 3 months after surgery, the patient reported significant improvement in tinnitus but continued to experience ear fullness. A total of 1 year after surgery, the patient reported a recurrence of intermittent loud, high-pitched, no pulsatile tinnitus not associated with quiet environment and persistent plugged ears.

## Discussion

SSWD is considered among the most frequent causes of venous anomalies. Dong *et al.* reported that SSWD accounts for 86.4% of the total vascular anomalies and variants associated with unilateral venous PT [11].

SS wall reconstruction for pulse synchronous tinnitus is a widely accepted and successful surgical solution [12, 13]. This can be achieved by reducing contact between the mastoid cells and the SS in several ways: (1) inserting bone chips between the bone wall structure and the SS to avoid direct contact with the mastoid cells. (2) Reinforcing the bone wall to reduce the amount of sound transmission caused by disruption of laminar flow in the SS. (3) Simple mastoidectomy to reduce sound conduction by mastoid cells. Endovascular interventions have been used to prevent the transmission of audible turbulence to mastoid air cells caused by the loss of blood laminar flow, primarily in patients presenting with aneurysms or diverticula, as our group has previously described [10]. However, we report three patients with SSWD without aneurysms or diverticula treated with an SS resurfacing stenting technique.

The pathophysiology of the associations of SS wall anatomical anomalies and their relation to the origin of PT are not well understood. Theories include vein blood flow dynamics, intracranial hypertension, and associated osteoporosis. Disruption of laminar vein blood flow may cause audible local turbulence, or alterations in the morphological inner ear anatomy may predispose individuals to increased bone conduction of normal blood flow [14].

Contralateral and ipsilateral stenosis of the transverse sinus has been postulated as a possible underlying mechanism of tinnitus secondary to SSWD. An increased volume of blood directed to the dominant sinus with contralateral stenosis of the transverse sinus, or an increased velocity of blood flow driven to the ipsilateral stenosed sinus has been described as a cause of erosion of the bony wall adjacent to the sigmoid sinus, generating dehiscence and leading to tinnitus [14, 15].

PT associated with SS wall anomalies has traditionally been treated using surgical techniques, in which the main goal is to reconstruct the sigmoid sinus surface to its normal smooth contour. This approach aims to eliminate vein flow turbulence and decrease the transmission of vibrations through mastoid air cells into the cochlea [16]. For novel endovascular strategies, coils and stents are used to decrease blood flow turbulence under the same resurfacing principle, reinforcing the vein wall.

Resurfacing techniques for the SS wall appear to be effective and well tolerated for most patients with bothersome PT caused by venous vascular abnormalities. Liu *et al.* reported a 75% total resolution of PT in patients treated with surgical resurfacing techniques focused on an SS source [3], with a 4% long-term morbidity and mortality rate. Endovascular procedures targeting pulsatile tinnitus associated with SS anomalies have been increasingly used in recent years. In 2017, Wang *et al.* showed that only 7.9% of patients (ten patients) were treated endovascularly [4]. However, excellent outcomes were reported, with a 91% pulsatile tinnitus resolution rate and only one case of small cerebellar ischemia, which showed neurological recovery 2 months after the procedure. In our small series, no long-term neurological morbidity or mortality was documented. Further endovascular data analysis will provide a better understanding of pulsatile tinnitus pathophysiology and allow for proper comparisons of the risks and outcomes between endovascular and surgical resurfacing techniques.

## Conclusion

Cannulation of SS through a durotomy in the SSS seems to be effective and safe when the jugular bulb is not favorable for stent navigation to the SS. Stenting in patients with SSWD not associated with diverticulum is an effective alternative treatment option for otherwise untreatable pulsatile tinnitus. Based on the data for venous stenosis stenting in the treatment of idiopathic intracranial hypertension, contralateral transverse sigmoid sinus stenting could be evaluated in the future to indirectly decrease the ipsilateral flow to the SSWD associated with PT.

## Data Availability

Not applicable.

## Abbreviations

SS: Sigmoid sinus
SSWD: Sigmoid sinus wall dehiscence
PT: Pulsatile tinnitus
SSS: Superior sagittal sinus
SSW: Sigmoid sinus wall
IJV: Internal jugular vein
CTA: Computed tomography angiography
